# Manifest/Non-Manifest Drug Release Patterns from Polysaccharide Based Hydrogels—Case Study on Cyclodextrin—κ Carrageenan Crosslinked Hydrogels

**DOI:** 10.3390/polym13234147

**Published:** 2021-11-27

**Authors:** Elena Simona Băcăiță, Cătălina Anișoara Peptu, Corina-Lenuta Savin (Logigan), Marian Luțcanu, Maricel Agop

**Affiliations:** 1 Department of Physics, Faculty of Machine Manufacturing and Industrial Management, “Gheorghe Asachi” Technical University of Iasi, Bd. Prof. Dr. Docent Dimitrie Mangeron 73, 700050 Iasi, Romania; elena-simona.bacaita@academic.tuiasi.ro (E.S.B.); maricel.agop@academic.tuiasi.ro (M.A.); 2 Department of Natural and Synthetic Polymers, Faculty of Chemical Engineering and Environmental Protection, “Gheorghe Asachi” Technical University of Iasi, 71, Prof. Dr. Docent Dimitrie Mangeron Street, 700050 Iasi, Romania; savincorina@yahoo.com; 3 Materials Science Department, Faculty of Materials Science and Engineering, “Gheorghe Asachi” Technical University of Iasi, 71, Prof. Dr. Docent Dimitrie Mangeron Street, 700050 Iasi, Romania; marian.lutcanu@staff.tuiasi.ro

**Keywords:** β cyclodextrin, κ carrageenan, films, hydrogels, epichlorohydrin, drug delivery, fractal, non-manifest

## Abstract

The aim of this study is to offer a comprehensive view on drug release from hydrogel, from both an experimental and a theoretical point of view. Aiming to benefit cyclodextrins’ properties (not irritant; stable; able to modify the physical, chemical and biological properties of active compounds; accessible at low prices) and those of carrageenan polysaccharide (antitumor, immunomodulatory, antihyperlipidemic, anticoagulant, biocompatibility, biodegradability), original hydrogel films based on beta cyclodextrin and kappa carrageenan using epichlorohydrin as crosslinking agent were prepared and characterized from morphological and physical/chemical points of view. The results (morphology, the swelling degree, and the loading/release capacity) proved their potential as carriers for different types of drugs. Further, a new theoretical model, from a multifractal paradigm of motion, was proposed for the drug release from hydrogel films, starting from the fundaments of its evolution at a microscopic level, and aiming to obtain information on system evolution, at both the spatial and temporal scales, inapproachable by quantitative measurements.

## 1. Introduction

Films can be manufactured basically from different materials, such as various resins, various synthetic polymers—polypropylene, polystyrene, polycarbonate, styrene acrylonitrile copolymer, and poly(methyl methacrylate) [1,2], respectively—and natural polysaccharides such as carrageenan, alginates, carboxymethyl cellulose, gellan, and pullulan. Considering the variety of materials used for the preparation of polymeric films, these can be transparent, semitransparent, opaque, colored, or smooth. Also, polymeric films present the capacity to absorb large amounts of water without dissolving, due to physical and chemical crosslinking processes between individual polymer chains. Moreover, polymeric films present excellent unique properties such as the ability to include biomacromolecules, biocompatibility/bioresorbability, the ability to be designed to be sensitive to various environmental factors (i.e., pH, temperature, and light), easy to prepare into various shapes and sizes, and with porous structure, all properties that recommend them as suitable platforms for drug delivery systems and medical devices [3,4]. Modern medicine uses a variety of natural and/or synthetic polymeric materials. Cyclodextrins (CD) have been extensively investigated in the medical field because of their numerous advantages, such as stability, not being an irritant, and their ability to modify physical, chemical and biological properties of active compounds through the formation of inclusion complexes, including the reduction in volatility of the drug molecule, and, just as importantly, their accessibility at low prices [5,6]. However, their use as a film component involves some limitations, like low water absorption capacity, high molecular weight, and possible parenteral toxicity, which limits the amount of CD to be used. To overcome these limitations, an alternative is to add a polysaccharide to the polymeric matrix [6,7,8].

Carrageenan (CG) is an anionic, hydrophilic, sulfated natural polysaccharide, extracted from red algae, with a high molecular weight of over 100 kDa, formed by a disaccharide repeating unit which consists of alternating 3-linked β-d-galactopyranose or 4-linked α-d-galactopyranose or 4-linked 3,6-anhydro-α-d-galactopyranose [8,9]. There are three major types of CG depending on the number and the position of sulphate groups per repetitive disaccharide unit corresponding to ideal structures, namely Kappa (κ)-, Iota (τ)-, and Lambda (λ)- carrageenan [10,11]. CG presents different pharmaceutical properties, such as antitumor, immunomodulatory, antihyperlipidemic, and anticoagulant activity [12]. Moreover, their biocompatibility, biodegradability, and water uptake determined the expansion of their use in different applications. For example, they are widely used as excipients in various drug delivery systems, such as controlled drug release systems since they are pH/temperature sensitive in contact with the physiological media of ophthalmic, oral, and vaginal drugs [13].

Aiming to benefit the cyclodextrin and carrageenan properties previously mentioned, original hydrogel films based on beta cyclodextrin and kappa carrageenan using epichlorohydrin were prepared and characterized in order to determine their swelling capacity and loading/release drug ability. All materials were prepared using epichlorohydrin (ECH) as a crosslinker agent under alkaline conditions [14]. Their structures were characterized by Fourier transform infrared spectroscopy (FT-IR) and scanning electron microscopy (SEM); the drug-loading and drug-releasing properties for all obtained films, using metronidazole (MT) as a model drug, were investigated.

The release kinetics were analyzed and interpreted, also, through theoretical model, which considers the system formed by the hydrogel film, the drug and the release environment a complex one, due to huge number of interactions taking place within. Such a system is very difficult to analyze in the hypotheses of classical physics, which is the reason why we propose a new approach: replace the system complexity with the concept of fractality. The theoretical model will offer insights on the system evolution at a microscopic scale and, further, by fitting with the experimental results, system-specific characteristic quantities such as the diffusion coefficient and Higuchi constant (as a measure of release rate) will be determined.

## 2. Experimental Procedure

### 2.1. Materials

The following materials were used for the experiments: βCD, κCG, NaOH, and ECH, which were purchased from Sigma-Aldrich (St. Louis, MO, USA); Metronidazole 99% which was purchased from Alfa Aesar (Haverhill, MA, USA); Milli-Q ultrapure distilled water from Merck Chemicals (Darmstadt, Germany). Epichlorohydrin (ECH) was used as a crosslinking agent without any pretreatment. The chemical reagents used were of analytical grade purity and were used without further purification.

### 2.2. Instrumentation

Films’ morphology was investigated via the SEM technique. SEM images were recorded with a Hitachi SU 1510 Scanning Electron Microscope (Tokyo, Japan); films were fixed on an aluminum stub and coated with a 7 nm thick gold layer using a Cressington108 device before observation. All SEM images were visualized and recorded at an accelerating voltage of 25 kV.

The Fourier transform infrared spectroscopy spectra of films were recorded with a Varian Digilab Scimitar FTS 2000 spectrometer (Crawley, United Kingdom). The samples were prepared as KBr pellets and scanned over the wave number range of 4000–450 cm^−1^ at a resolution of 4.0 cm^−1^.

### 2.3. Methods

#### 2.3.1. Preparation of βCD-CG Films

Films were synthesized via the Jeong et al. method with some modifications, and the reactant composition is shown in Table 1. βCD/κCG films were prepared as follows: 500 mg of κCG was dissolved in different volumes of 9 wt.% NaOH solution (10, 15, 20 mL). Next, 500 mg of βCD was added, and 1.5 mL of ECH was slowly added to the solution while stirring at 600 rpm at 25 °C for 20 min. The resulting films were washed with ethanol solution. Finally, all the samples were lyophilized. The film yield was obtained using the following equation:

$$Films\;yield,\;\%\;=\;\frac{Dry\;weight\;of\;film}{Total\;weight\;of\;reactant\;in\;feed}\;\times 100$$

#### 2.3.2. Evaluation of Swelling Characteristic

Swelling studies were performed by gravimetric method. Briefly, all freeze-dried films were weighted and immersed in vials (glass vial, 10 mL) containing 3 mL of DW. The vials were maintained at room temperature for 96 h. The swelling process was monitored, and at different times, DW was removed and films were weighed; the remaining water on the surface was gently blotted away with laboratory tissues.

The water swelling ratio was determined with the equation:

$$Q(\%)\;=\;\frac{w_{s}\;-\;w_{0}}{w_{0}}\;\times 100$$

where $w_{s}$ is the weight of the swollen probe, and $w_{0}$ is the weight of the dry probe.

#### 2.3.3. Evaluation of Metronidazole Loading and Release

Drug loading studies were performed via a diffusion mechanism. In this regard, MT was used as model drug. Briefly, dried films were placed in a glass vial (10 mL) in 3 mL of MT aqueous solution (10 mg/mL). The films were maintained at room temperature. The amount of MT loaded in films was spectrophotometrically determined based on a calibration curve previously obtained. After 48 h, the MT-loaded films were gently washed with DW, and the water on the surface of the films was gently removed with a laboratory tissue.

The release profiles of the MT from films were evaluated in DW at room temperature. At different times, 20 µL of volume-releasing solution were withdrawn from the DW. The volume of DW was held constant by adding fresh DW. The cumulative amount of MT was determined via the spectrophotometrical method. All measurements were performed in triplicate and averaged. All tests were accomplished with a spectrophotometer UV–VIS NanoDrop ND-1000, which allows for the analysis of very small sample volumes, in the microliter range. The loaded/released drug quantity was determined by monitoring the wavelength at 320 nm for MT.

## 3. Results and Discussion

Films, based on βCD and κCG, with ECH as a crosslinking agent, were prepared. In order to obtain information about the βCD/κCG network, first, different molar ratios between βCD and κCG were varied, with respective ECH concentrations, as well as NaOH reaction volume. It was found that 0.02 mol of ECH led to an excellent crosslinked network while maintaining the good integrity of the film. Therefore, for all further studies, the βCD/κCG films were prepared in the presence of 0.02 mol ECH.

Moreover, the results (Table 1) revealed that the best film obtained had the following reaction parameters: a reaction time of 20 min, an ECH concentration of 0.02 mol with respect to the total amount of polymers, and a low reaction volume of 10 mL. In consequence, next, a 1:1 molar ratio (βCD/κCG), 0.02 mol ECH and 10 mL NaOH 9 wt.% were used.

After the purification with ethanol and freeze drying, the determined βCD/κCG films recovered fractions were found to depend on the polymer concentration. Therefore, the yields of films were dependent on the polymer concentration: higher in the case of a high polymer concentration and lower when the polymer concentration was decreased, as shown in Table 1.

### 3.1. Structural Characterization

#### 3.1.1. FTIR Characterization

**Figure 1:**
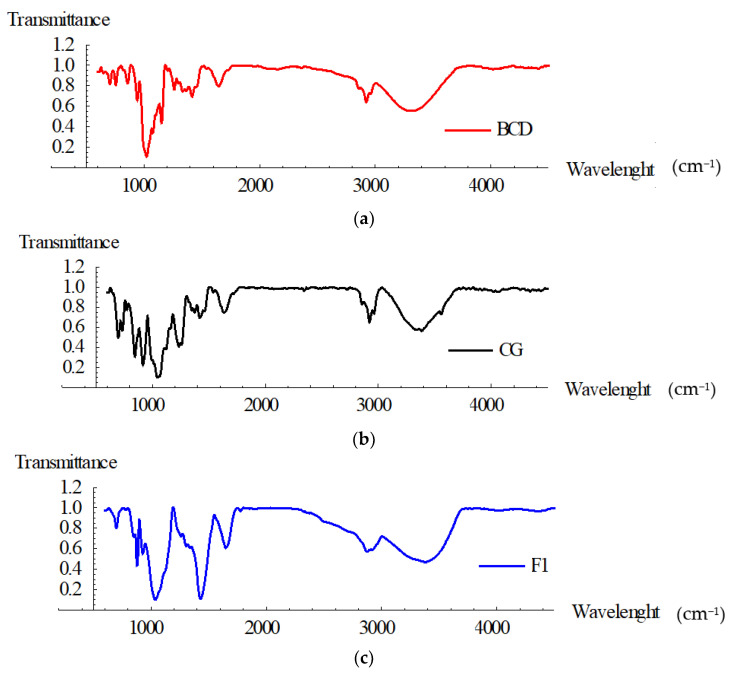
FTIR spectra of the βCD (**a**), κCG (**b**), and F1 crosslinked film synthesized (**c**).

In accordance with the theoretical reaction mechanism, carboxylate and hydroxyl groups of both polymers attack either the epoxide or CHCl_2_ groups of ECH in order to form ester bonds. These linkages were certified via FT-IR spectra of the βCD, κCG and the crosslinked film synthesized analogy (Figure 1).

The spectra of βCD is characterized by an intense band at 3302 cm^−1^ due to O–H stretching vibration, while the vibration of the –CH and –CH_2_– groups appears in the 2800–3000 cm^−1^ region. The κCG spectra showed the hydroxyl groups (O–H) absorption peak appeared at 3384 cm^−1^ in with high intensity. Also, κCG bands observed at 844, 916, 1043, and 1234 cm^−1^ can be attributed to d-galactose-4-sulfate, 3,6-anhydro-d-galactose, glycosidic linkage and ester sulfate stretching.

The bands observed at 1649, 1257, and 1035 cm^−1^ in the film spectra can be attributed to stretching modes of (C=O), (C–C–O), and (O–C–C) bonds of the ester group, respectively. Moreover, at 1429 cm^−1^ was observed the appearance of an intense peak O–H bending compared to spectra of crude βCD and κCG, which confirms that the synthesized film is crosslinked.

#### 3.1.2. Morphological Characterization by SEM

**Figure 2:**
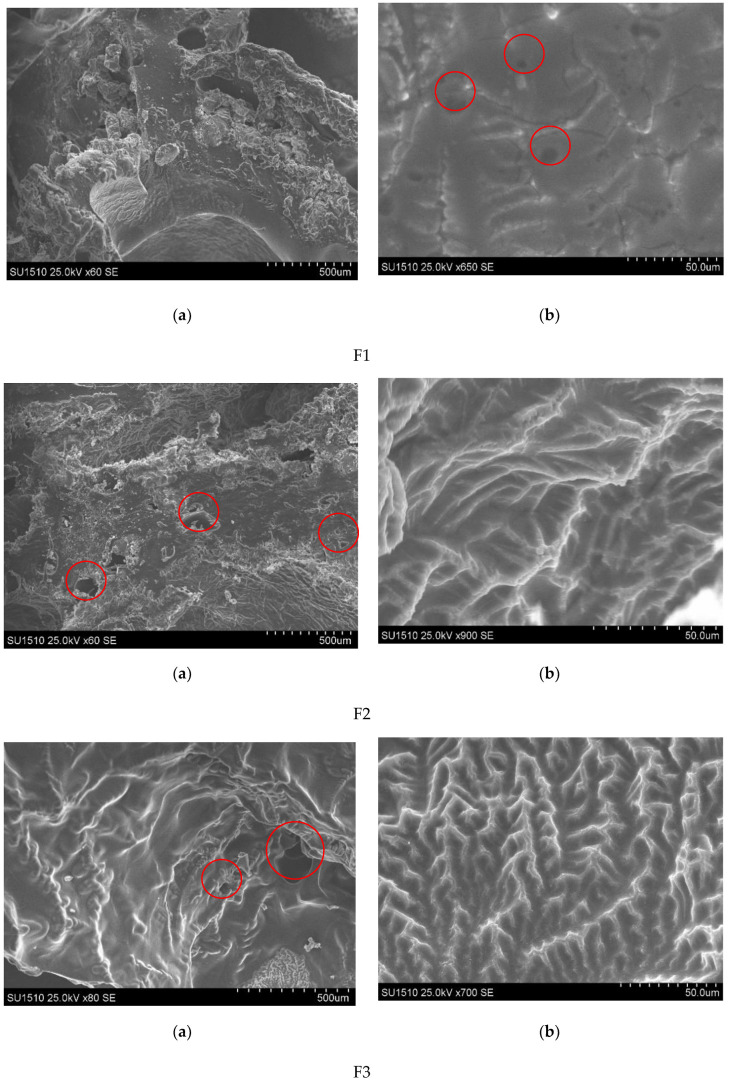

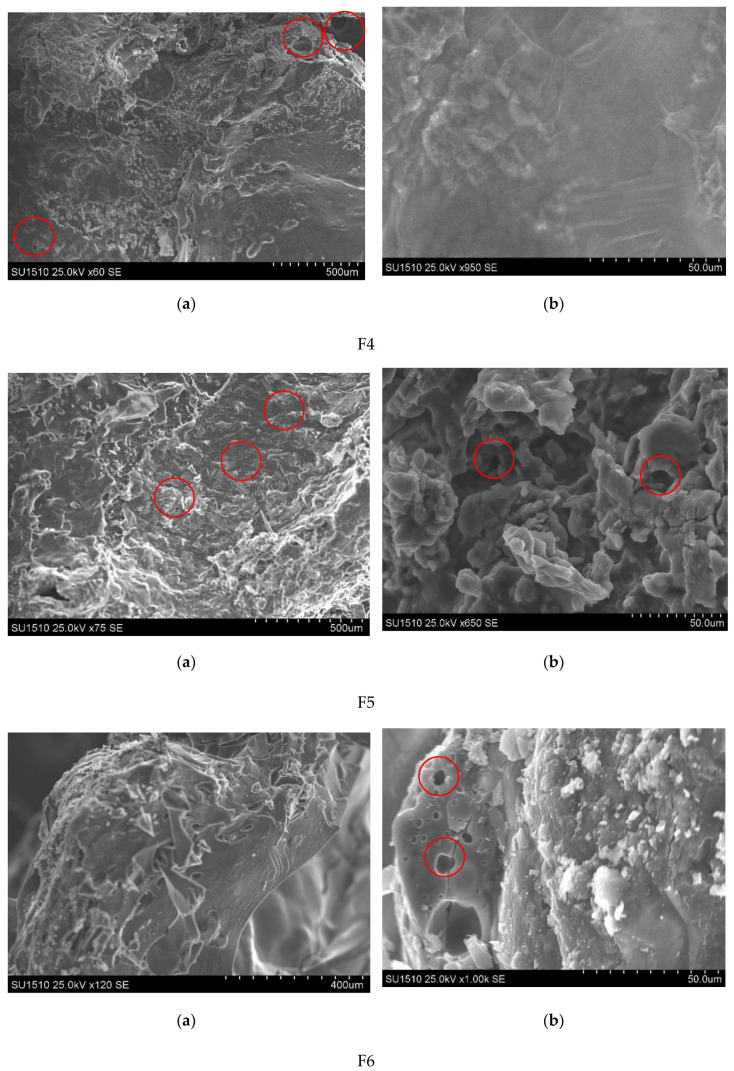

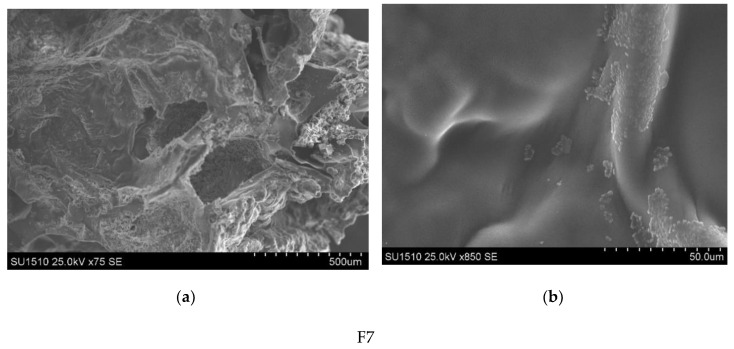
SEM images of the βCD/κCG films at various magnifications (scale bar length: (**a**) 0.5 mm and (**b**) 0.05 mm) for all films F1–F7.

One of the most important properties that must be considered is the film structure morphologies. SEM technique was used to visualize the morphological characteristics of lyophilized polymeric films. When comparing the films’ images, was found that all films are relatively compact, with a surface which presents roughness and some aggregates. However, a closer look at the cross-section (Figure 2b at scale bar length 0.05 mm) revealed also some areas with pores in the samples F1, F2, F3 and F5, F6 (see the enclosed areas in SEM images). The porosity decreases when the ECH concentration was increased. Furthermore, SEM images of sample F7 revealed that it has a smooth surface with reduced porosity, which may explain the film reduced water uptake capacity.

### 3.2. Evaluation of Hydrogels Behavior in Aqueous Media

**Figure 3:**
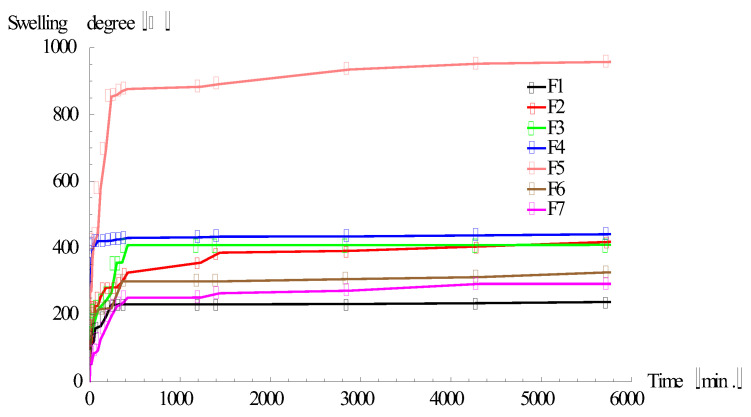
Films swelling ratio.

In order to predict and comprehend the βCD/κCG films’ ability to encapsulate/release drugs, a further characterization was performed by analyzing an important factor which is determinant, namely the swelling properties. Swelling studies were performed by gravimetric method and the swelling degree of samples in DW is shown in Figure 3. The results revealed a correlation between the swelling degree and films’ preparation parameters, such as polymer concentration, reaction volume, and crosslinking agent ECH concentration. The sample which showed a high superabsorbent property is sample F5 (1:1 [βCD:κCG] molar ratio and 5% polymer concentration), namely 957%. The higher water uptake for the F5 sample, obtained ata lower polymer feed concentration, is as we expected, since at lower concentrations a rather looser network is formed. As we can see in Table 2, the swelling results are dependent on the polymer and crosslinking agent concentration. We can state that the films’ water absorbencies are decreasing with the increasing of the polymer and ECH concentration. Also, the decreased swelling ratio of the films can be attributed to the βCD, which presents hydrophobicity and rigidity, thus influencing the flexibility of the polymer network, respectively the water uptake capacity. Nevertheless, considering the presence of βCD in the polymer network, the main reason for a good water uptake in the films may be determined by the presence of sulfate groups in its κCG parts.

### 3.3. Evaluation of Metronidazole Loading and Release Kinetics

The possibility of using βCD/κCG materials as drug delivery systems was also investigated. The drug loading and release characteristics of the films were examined using metronidazole (MT) as model drug; it is a limited spectrum antibiotic that actively deters the growth of protozoa, anaerobic gram-positive, and anaerobic gram-negative bacteria. Therefore, all samples were loaded with MT solution and, subsequently, the released MT in DW was measured.

The results, summarized in Table 3, showed that the MT amount loaded into films after 48 h, varied between 10.5 and 17.9 mg. Also, as in the swelling analysis, the quantity of MT loaded proved to be dependent on the polymer and crosslinking agent concentration. As we expected, the F5 sample, with a high-water uptake, demonstrated also a high drug-loaded capacity (17.9 mg MT); this result may be explained by the fact that the sample F5 had a stronger complexation capability compared to sample F7. In terms of efficiency, it varied between 40% and 65%.

**Figure 4:**
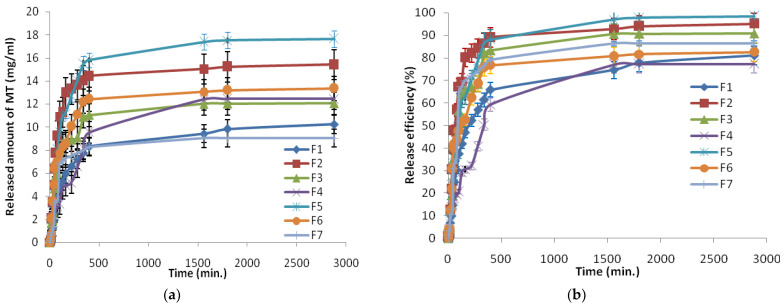
The release kinetics in terms of mg/mL (**a**) and release efficiency (**b**).

In order to evaluate the MT release, MT-loaded films were added into the release medium and the release kinetics are represented in Figure 4, in terms of mg/mL (Figure 4a) and MT released efficiency (Figure 4b). The results showed an initial burst release phase, within the initial approximately 30 min, followed by a slower release phase, characterized by a final constant release, until 48 h. By analyzing the influence of preparation parameters on the release ability of the films, similar behavior as for MT loading was observed. The maximum MT amount released varied between 9.03 and 17.65 mg/mL, as can be seen in Table 3, which also enables a comparative analysis between loaded and released MT.

Overall, the efficiency of MT release for all analyzed films had values between 80% and 98%. As anticipated, the highest amounts of MT were released from the F5 sample (17.65 mg/mL), which has been shown to have the highest water uptake. These results indicate that the sample F5 is more effective as a drug delivery system, being capable of encapsulating a large amount of MT and releasing it effectively. All MT release profiles demonstrated that βCD/κCG films can be promising and attractive materials for controlled drug delivery systems in a variety of applications.

## 4. Patterns in Drug Release Phenomena

As previously mentioned, the proposed theoretical model is built on the idea of assimilating the system complexity with the concept of fractality. An intuitive way to understand fractality is by looking inside of the complex system at microscopic level: between two successive “events”the trajectory of a system structural unit (which can be either water/drugmolecule or hydrogel fragment) is a straight line, but due to the system complexity, these “events” form an uncountable set of points in the space-time coordinates that define a three dimensional fractured line, named fractal curve, whose non-linearity is characterized through a quantity named fractal dimension; obviously, its value depends on the scale resolution, being higher for smaller scale resolutions. If the system is inhomogeneous and anisotropic, as, actually, is the studied system, areas with different fractal dimensions appear simultaneously, in which case we will state that the trajectory of the system’s structural unit follows a multifractal curve, characterized by multifractal dimensions [15,16,17].

In these assumptions, we are justified in assuming that the structural units of the complex system move on fractal/multifractal curves that are continuous, but non-differentiable. From such a perspective, instead of working with a single variable (i.e., the amount of released drug), we will operate with approximations of this function, i.e., the mathematical function given by averaging them on various scale resolutions. So, it results that the mathematical variable proposed to characterize the release kinetics will act as the limit of a class of functions variable and will be non-differentiable for null scale resolutions and differentiable otherwise [18]. From such a perspective, in the framework of the scale relativity theory [15,16,17], the release kinetics will be assimilated with the flow regimes of a multifractal fluid (turbulent regime, laminar regime, etc.), as will be shown in the following.

Therefore, let us consider the scale covariance derivative, as motion operator, in the description of the system structural units dynamics [17,18]:

(1) $$\frac{\overset{\wedge }{d}F}{dt}\;=\;\left[\partial_{t}\;+\;\overset{\wedge }{V^{l}}\partial_{l}\;+\;\frac{1}{4}{\left(dt\right)}^{{}^{\left[\frac{2}{g\left(\alpha \right)}\right]-1}}D^{rp}\partial_{r}\partial_{p}\right]F$$

where

$$\overset{\wedge }{V^{r}}\;=\;V_{D}^{r}\;-\;V_{F}^{r}$$

$$D^{rp}\;=\;d^{rp}\;-\;i\overset{\wedge }{d^{rp}}$$

(2) $$d^{rp}\;=\;\lambda_{+}^{r}\lambda_{+}^{p}\;-\;\lambda_{-}^{r}\lambda_{-}^{p}$$

$$\overset{\wedge }{d^{rp}}\;=\;\lambda_{+}^{r}\lambda_{+}^{p}\;+\;\lambda_{-}^{r}\lambda_{-}^{p}$$

$$\partial_{t}\;=\;\frac{\partial }{\partial t};\partial_{r}\;=\;\frac{\partial }{\partial x^{r}};\partial_{r}\partial_{p}\;=\;\frac{\partial }{\partial x^{r}}\frac{\partial }{\partial x^{p}};i\;=\;\sqrt{-1};r,p\;=\;1,2,3$$

and

- F is a multifractal function;
- $x^{r}$ is the multifractal spatial coordinate;
- t is the non-multifractal time coordinate, also playing therole of an affine parameter of the trajectories, meaning that the analysis of release dynamics is done from the perspective of a projective geometry;
- dt is the scale resolution;
- $\overset{\wedge }{V^{r}}$ is the complex velocity of system structural unit;
- $V_{D}^{r}$ is the differentiable velocity of system structural unit—independent on dt,
- $V_{F}^{r}$ is the non-differentiable velocity of system structural unit—dependent on dt,
- $D^{rp}$ is a constant tensor, corresponding to the non-differentiable—differentiable scale transition (i.e., transitions from the microscopic to the macroscopic scale in the release dynamics);
- $\lambda_{-}^{r}\left(\lambda_{-}^{p}\right)$ and $\lambda_{+}^{r}\left(\lambda_{+}^{p}\right)$ are constant vectors corresponding to the backward and forward non-differentiable—differentiable drug release dynamics, through which the release dynamics can be explained by transitions from the microscopic to the macroscopic scale;
- and $g\left(\alpha \right)$ defines the singularity spectrum of order α, where α is the singularity index and is a function of fractal dimension $D_{F}$ in the form $\alpha \;=\;\alpha \left(D_{F}\right)$ [19,20].

The multifractal analysis [21], through the singularity spectrum, reveals the existence of the following “release patterns”:

(i) monofractal release patterns, which implies release in a homogeneous system, characterized through a single fractal dimension and having the same scaling properties in any time interval;
(ii) multifractal release patterns, which include release in an inhomogeneous and anisotropic system, characterized simultaneously by a wide variety of fractal dimensions.

Thus, $g\left(\alpha \right)$ allows the identification of universality classes in the field of dynamic release systems, even when the strange attractors of the release dynamics have different aspects.

### 4.1. Release Dynamics as Turbulent or Laminar Flows of a Multifractal Type Fluid

Now, accepting the functionality of the scale covariance principle, according to which release processes are governed by invariant physical laws relative to scale resolutions, and, by applying the operator (1) to the complex velocity from (2), without any restraints, the motion equation of any complex system structural unit can be written in the form:

(3) $$\frac{d\overset{\wedge }{V^{i}}}{dt}\;=\;\partial_{t}\overset{\wedge }{V^{i}}\;+\;\overset{\wedge }{V^{r}}\partial_{r}\overset{\wedge }{V^{i}}\;+\;\frac{1}{4}{\left(dt\right)}^{{}^{\left[\frac{2}{g\left(\alpha \right)}\right]-1}}D^{rp}\partial_{r}\partial_{p}\overset{\wedge }{V^{i}}\;=\;0$$

where $\partial_{t}\overset{\wedge }{V^{i}}$ represents local multifractal acceleration, $\overset{\wedge }{V^{r}}\partial_{r}\overset{\wedge }{V^{i}}$ represents multifractal convection, and $D^{rp}\partial_{r}\partial_{p}\overset{\wedge }{V^{i}}$ represents multifractal dissipation. Therefore, at any point of the release path, multifractal inertia, multifractal convection, and multifractal dissipation make their balance.

In these conditions, separating the complex system structural unit dynamics on scale resolutions, both at differentiable and non-differentiable scale resolutions, Equation (3) can be dissociated in two equations:

(4) $$\begin{array}{l} \partial_{t}V_{D}^{i}\;+\;V_{D}^{r}\partial_{r}V_{D}^{i}\;-\;V_{F}^{r}\partial_{r}V_{F}^{i}\;+\;\frac{1}{4}{\left(dt\right)}^{{}^{\left[\frac{2}{g\left(\alpha \right)}\right]-1}}D^{rp}\partial_{r}\partial_{p}V_{D}^{i}\;=\;0\\ \partial_{t}V_{F}^{i}\;+\;V_{F}^{r}\partial_{r}V_{D}^{i}\;+\;V_{D}^{r}\partial_{r}V_{F}^{i}\;-\;\frac{1}{4}{\left(dt\right)}^{{}^{\left[\frac{2}{g\left(\alpha \right)}\right]-1}}D^{rp}\partial_{r}\partial_{p}V_{F}^{i}\;=\;0 \end{array}$$

which reflects the fact that drug release involves interdependent complex mechanisms, both at differential and non-differential scale resolution.

Both in Equation (3) at global scale resolutions as well as in Equation (4) at differential and non-differential scale resolutions, the multifractalization procedure is not made explicit. Since for a large temporal scale resolution, with respect to the inverse of the highest Lyapunov exponent [20,21], the deterministic trajectories of any system structural unit can be substituted with virtual trajectories, the concept of definite trajectory can be replaced by one of probability density [20,21]. In such a perspective, the multifractalization through stochasticization becomes functional in describing the release system through the dynamics of a multifractal type fluid. Therefore, in the following, we shall consider only the case of multifractalization by Markov-type stochastic processes, which imply the conditions [16,19,20,21]:

(5) $$\begin{array}{c} \lambda_{+}^{i}\lambda_{+}^{r}\;=\;\lambda_{-}^{i}\lambda_{-}^{r}\;=\;\lambda \left(\mu \right)\delta^{ir}\\ \mu \;=\;{\left(dt\right)}^{{}^{\left[\frac{2}{g\left(\alpha \right)}\right]-1}} \end{array}$$

where $\lambda \left(\mu \right)$ are specific coefficients associated to the multifractal–non-multifractal scale transition, and $\delta^{ir}$ is the Kronecker’s pseudo-tensor. In the present context, these scale transitions can be made explicit by changing the diffusion regimes, for example, from Fickian-type to non-Fickian-type diffusions. We note that stochasticization by Markov-type processes is a multifratalization procedure often used in physics; some of the consequences of this type of stochasticization are the usual Fickian diffusion and the Nelson procedure for obtaining the Schrödinger equation [16,17,18,19,20,21].

In conditions expressed by (5), the Equation (3) becomes:

(6) $$\frac{d\overset{\wedge }{V^{i}}}{dt}\;=\;\partial_{t}\overset{\wedge }{V^{i}}\;+\;\overset{\wedge }{V^{r}}\partial_{r}\overset{\wedge }{V^{i}}\;-\;i\lambda \left(\mu \right)\partial_{r}\partial^{r}\overset{\wedge }{V^{i}}\;=\;0$$

in which case the separation of the release dynamics on scale resolutions implies the functionality of the following differential equations for the velocity fields:

(7) $$\begin{array}{l} \partial_{t}V_{D}^{i}\;+\;V_{D}^{r}\partial_{r}V_{D}^{i}\;-\;\left[V_{F}^{r}\;+\;\lambda \left(\mu \right)\partial^{r}\right]\partial_{r}V_{F}^{i}\;=\;0\\ \partial_{t}V_{F}^{i}\;+\;V_{D}^{r}\partial_{r}V_{F}^{i}\;+\;\left[V_{F}^{r}\;+\;\lambda \left(\mu \right)\partial^{r}\right]\partial_{r}V_{D}^{i}\;=\;0 \end{array}$$

In such an alternative, the release dynamics can be assimilated to turbulent flows of the multifractal type fluid.

For irotational movements of system structural units, the complex velocity field (2) becomes:

(8) $$\overset{\wedge }{V^{i}}\;=\;-2i\lambda \left(\mu \right)\partial^{i}\ln\Psi$$

with Ψ the state function. If Ψ has the form (the Madelung’s type choice):

(9) $$\Psi \;=\;\sqrt{\rho }e^{is}$$

where $\sqrt{\rho }$ can be considered amplitude and s the phase, the complex velocity fields (8) transform into:

(10) $$\overset{\wedge }{V^{i}}\;=\;2\lambda {\left(dt\right)}^{{}^{\left[\frac{2}{g\left(\alpha \right)}\right]-1}}\partial^{i}s\;-\;i\lambda {\left(dt\right)}^{{}^{\left[\frac{2}{g\left(\alpha \right)}\right]-1}}\partial^{i}\ln\rho$$

which leads to the determination of velocity fields:

(11) $$\begin{array}{l} V_{D}^{i}\;=\;2\lambda \left(\mu \right)\cdot \partial^{i}s\\ V_{F}^{i}\;=\;\lambda \left(\mu \right)\cdot \partial^{i}\ln\rho \end{array}$$

In such an alternative the release dynamics can be assimilated to laminar flows of the multifractal fluid, the state function Ψ having physical significance only in the form $\rho \;=\;\Psi \bar{\Psi }$, where $\bar{\Psi }$ is the complex conjugated of Ψ, as probability density and, in particular, as the density of the system structural units.

By (11) and applying the mathematical approaches from [16,17], the Equation (7) become hydrodynamic equations of multifractal type:

(12) $$\partial_{t}V_{D}^{i}\;+\;V_{D}^{r}\partial_{r}V_{D}^{i}\;=\;-\partial^{i}Q$$

(13) $$\partial_{t}\rho \;+\;\partial_{r}\left(\rho V_{D}^{r}\right)\;=\;0$$

where with Q is denoted the specific potential of multifractal type:

(14) $$Q\;=\;-2\lambda^{2}\left(\mu \right)\frac{\partial^{r}\partial_{r}\sqrt{\rho }}{\sqrt{\rho }}\;=\;-V_{F}^{i}V_{F}^{i}\;-\;\frac{1}{2}\lambda \left(\mu \right)\partial_{r}V_{F}^{r}$$

The Equation (12) corresponds to the specific momentum conservation law of multifractal type, while the Equation (13) corresponds to the density conservation law of multifractal type. The potential Q expressed by (14) requires also a specific force of multifractal type:

(15) $$G^{i}\;=\;-2\lambda^{2}(\mu )\partial^{i}\frac{\partial^{r}\partial_{r}\sqrt{\rho }}{\sqrt{\rho }}$$

that quantifies the multifractality degrees of the motion trajectories. According to the previous results, the multifractality degrees of the motion trajectories can be mapped to the flow regimes of the multifractal fluid and thus, to the release dynamics of the complex system. Thus, the turbulent flows of the multifractal-type fluid can be associated with differentiable–nondifferentiable scale transitions in the release dynamics, while the laminar flows of the multifractal-type fluid can be associated with either a multifractal Fickian diffusion or a non-Fickian diffusion, etc.

### 4.2. Non-Manifest Release Patterns

In the following we will describe the dynamics of the complex polymer–drug system, accessing initially steady states, and then, through a harmonic mapping principle, non-stationary states. Through such an approach, various non-manifest patterns can be accessed. Since the force of multifractal type (15) acts as the “trigger” of the release process, we will consider first that the specific multifractal potential is constant. Then, the following equation in the one-dimensional case is satisfied:

(16) $$\begin{array}{l} \frac{d^{2}\sqrt{\rho }}{dx^{2}}\;+\;k^{2}\sqrt{\rho }\;=\;0\\ k^{2}\;=\;const./2\lambda^{2}(\mu ) \end{array}$$

The solution of this equation has the form:

(17) $$\sqrt{\rho }\;=\;ze^{i(kx\;+\;\Phi )}\;+\;\bar{z}e^{-i(kx\;+\;\Phi )}$$

where z is a complex amplitude, $\bar{z}$ is its complex conjugate and Φ is a specific phase. Thus, through z and Φ each structural unit of the system is labeled. Moreover, the multifractal group of SL(2R) type, defined as a hidden symmetry of Equation (16) through the parameters z, $\bar{z}$ and the unimodular factor τ [18,19,20,21],

(18) $$\begin{array}{l} z↔\frac{az\;+\;b}{cz\;+\;d}\\ \bar{z}↔\frac{a\bar{z}\;+\;b}{c\bar{z}\;+\;d};a,b,c,d\in R\\ \tau ↔\frac{c\bar{z}\;+\;d}{cz\;+\;d}\tau \end{array}$$

shows that this group can be assimilated with a “synchronization” group between various structural units of the complex system, a process in which participates, obviously, the amplitudes of each of them, in the sense that they are correlated, not only their phases. The “usual synchronization” manifested through the phase shift of the system structural units is, in this case, only a very particular case.

Now, accessing the non-stationary states of the complex system polymer-drug can be done in the space of $\left(z,\bar{z},\tau \right)$ variables, a space that can be structurated as a Riemann manifold of metric Poincaré [18,19,20,21]

(19) $$ds^{2}\;=\;-\frac{dz\times d\bar{z}}{{\left(z-\bar{z}\right)}^{2}}$$

based on a harmonic mapping principle.

Indeed, the function associated to the metrics (19),

(20) $$J\;=\;\frac{1}{2}\int \frac{\nabla z\nabla \bar{z}}{\left(z-\bar{z}\right)}d^{3}x$$

where ∇ denotes the gradient and $d^{3}x$ is the elementary volume implies, through the harmonic mapping principle [22,23,24,25,26].

(21) δJ = 0, 

the Euler Lagrange equations

(22) $$\begin{array}{l} {\left(z-\bar{z}\right)}^{2}\nabla^{2}z\;=\;2\nabla z\nabla z\\ {\left(z-\bar{z}\right)}^{2}\nabla^{2}\bar{z}\;=\;2\nabla \bar{z}\nabla \bar{z} \end{array}$$

The first from Equation (22) admits the solution:

(23) $$z\;=\;i\frac{\cosh\chi \;-\;e^{-i\alpha }\sinh\chi }{\cosh\chi \;+\;e^{-i\alpha }\sinh\chi }$$

with α real and χ a solution of a Laplace-type equation for the free space. Hence, for the choice α = ωt by which we specify the transition from stationary to non-stationary states, the Equation (23) with r = cothχ becomes:

(24) $$z\;=\;i\left[\frac{2r\sin(2\omega t)}{1\;+\;r^{2}\;+\;2r\cos(2\omega t)}\;+\;i\frac{1\;-\;r^{2}}{1\;+\;r^{2}\;+\;2r\cos(2\omega t)}\right]$$

**Figure 5:**
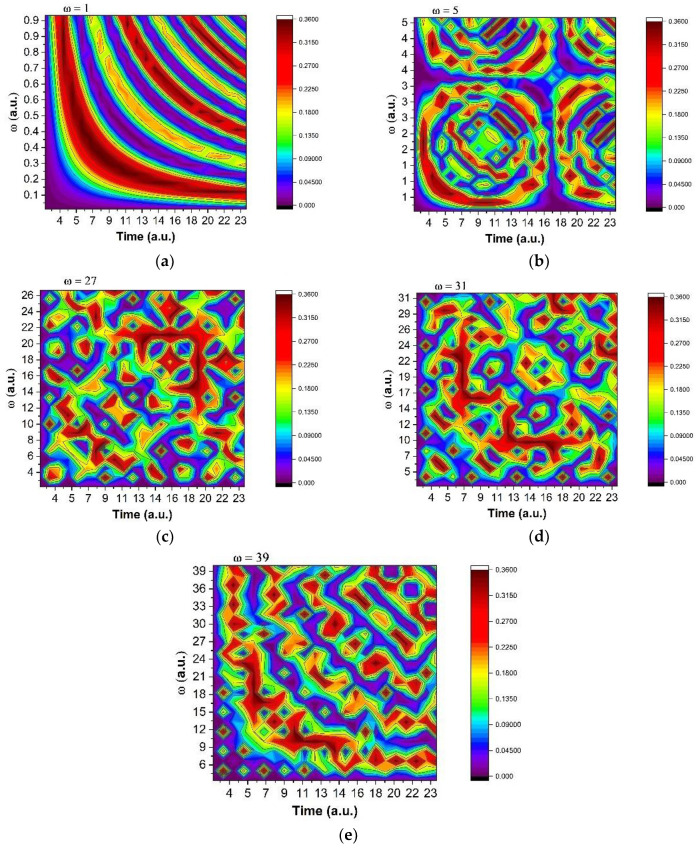
Contour plot of amplitude modulus of z for various interactions scales defined by *ω* factor: ω = 1 (**a**), ω = 5 (**b**), ω = 27 (**c**), ω = 31 (**d**), ω = 39 (**e**).

**Figure 6:**
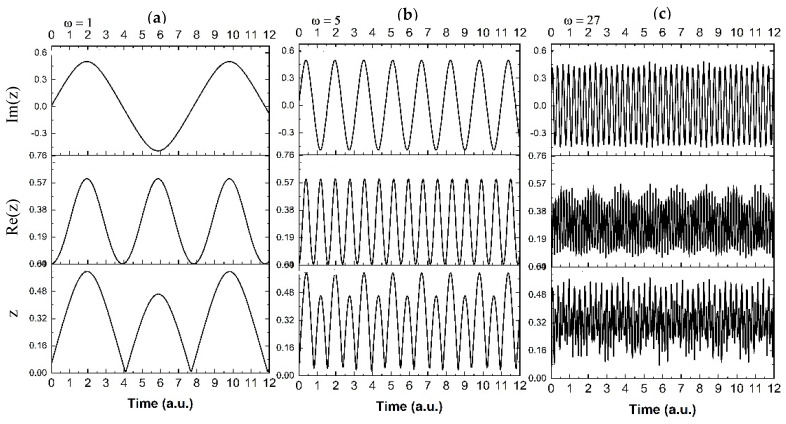

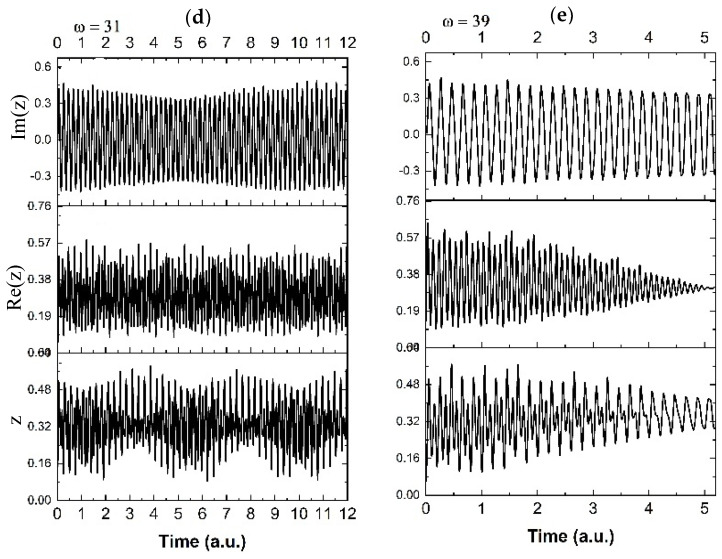
Time series of the oscillatory behavior for the z, Im(z), and Re(z) for various values of ω = 1 (**a**), ω = 5 (**b**), ω = 27 (**c**), ω = 31 (**d**), ω = 39 (**e**).

Since the “synchronization modes”, in phase and amplitude, of the complex system structural units imply group invariances of a SL(2R) type [27,28,29,30,31,32,33,34,35,36,37,38,39], then the period doubling emerges as a natural behavior in the complex system dynamics. In Figure 5 and Figure 6 we have represented the two-dimensional maps of the release function at various scales, defined by the *ω* parameter, which defines the interaction scale for which the function (25) defines drug release patterns. We observe that, for small scales (ω = 1), the system exhibits a burst release (Figure 5a) which is followed by a modulated release for ω = 5 (Figure 5b) ending with fractal-like structure for *ω* equal to or higher than 27 (Figure 5c–e).

To obtain better insights into the particularities of these fractal dynamics, we have extracted the temporal traces of *z*, *Im(z)* and *Re(z)* from the 2D maps characteristics of *ω* = 1, 5, 27, 31, 39. The results are presented in Figure 6. The use of the *Re* and *Im* part of *z* will allow us to differentiate between different contributions to the drug release. The imaginary part Im(z) of the function corresponds to the implicit release behavior which is hidden to the measurable values, but, on average, affects the nonlinear behavior of the release. The real part Re(z) will characterize the explicit measurable part of the release, i.e., the amount of released drug. From Figure 6 one can observe that, overall, the system evolves from a simple oscillation (Figure 6a) through a double period oscillatory type release (Figure 6b) towards a modulated release dynamic (Figure 6c). In the last phases of release, the system evolves finally to a cvasi-chaotic state (Figure 6d), ending with a damped oscillatory behavior (Figure 6e). The in/out jump from the cvasi-chaotic state to steady one implies that chaos is not specific to the release dynamics, while the other oscillatory type behaviors, i.e., simple, modulated, or damped oscillations, are more probable in the small-scale release scenarios [40,41,42,43,44,45,46,47].

Also, from Figure 6c–e, it can be observed that, for *ω* = 27, 31, 39, the main dynamic is given by the real part of the complex amplitude, since the modulation and the damped type dynamic seen in the *Re(z)* is seen also in *z*, the only difference happening in the oscillations frequency.

**Figure 7:**
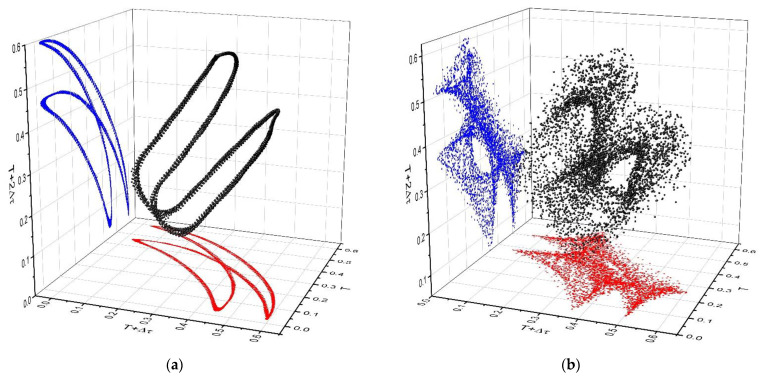

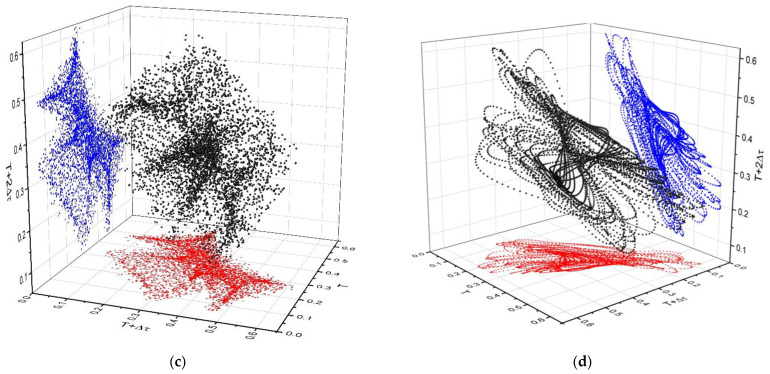
Reconstructed attractors for various local scenarios: period doubling for ω = 5 (**a**), modulation ω = 27,31 (**b**,**c**), and damped oscillation ω = 39 (**d**).

The strange attractors (Figure 7), which we will define as a non-manifest drug release pattern, specify the release mechanisms that occur and even if they are not macroscopic relevant, they still manifest imposing Fickian and non-Fickian type behaviours. As can be seen in Figure 7a, for the period doubling, the attractors have two main branches each characterizing one release frequency. The modulated attractors (Figure 7b,c) contain intermediary trajectories which define a three-dimensional attractor built on the scaffold of the period doubling one. When *ω* is high enough, drastic change in the attractor’s geometry occurs. Two lateral branches communicating with each other appear, resembling a Lorentz type attractor (Figure 7d).

### 4.3. One Example of Manifest Release Pattern

Since the release mechanisms imply correlations of the release dynamics at both scale resolutions, differentiable and non-differentiable, we will admit, according to [47,48], that the velocity fields $V_{D}^{i}$ and $V_{F}^{i}$ satisfy the condition:

(25) $$V_{D}^{i}\;=\;-V_{F}^{i}$$

In this condition, the conservation laws (12) and (13) integrate into a “diffusion equation” of multifractal type:

(26) $$\partial_{t}\rho \;=\;\lambda {\left(dt\right)}^{{}^{\left[\frac{2}{g\left(\alpha \right)}\right]-1}}\partial_{r}\partial^{r}\rho \;=\;\sigma \partial_{r}\partial^{r}\rho \sigma \;=\;\lambda {\left(dt\right)}^{\left[\frac{2}{g\left(\alpha \right)}\right]-1}$$

which suggests that the release process is a superposition of different diffusion types, i.e., Fickian, non-Fickian, taking place at different scale resolutions in a multifractal space.

Following the methodology from [48], for identical structure system, the solution is obtained in the form:

(27) $$f\;=\;\frac{\rho_{t}}{\rho_{\infty }}\;=\;2{\left(\frac{\sigma t}{d^{2}}\right)}^{\frac{1}{2}}\;=\;\left\{\pi^{-1/2}\;+\;\sum_{n\;=\;1}^{\infty }{\left(-1\right)}^{n}erfc\left[\frac{nd}{2{\left(\sigma t\right)}^{\frac{1}{2}}}\right]\right\}$$

For small time scales, reachable through experiments, the second term of (27) disappears and it leads to relation:

(28) $$\frac{\rho_{t}}{\rho_{\infty }}\;=\;2{\left(\frac{\sigma t}{d^{2}}\right)}^{\frac{1}{2}}\;=\;const\times t^{\frac{1}{2}}\Leftrightarrow \frac{M_{t}}{M_{\infty }}\;=\;2{\left(\frac{\sigma t}{d^{2}}\right)}^{\frac{1}{2}}\;=\;const\times t^{\frac{1}{2}}$$

which is actually the Higuchi equation, $\frac{M\left(t\right)}{M\left(\infty \right)}\;=\;k_{H}t^{\frac{1}{2}}$, one of the most known equations used in modeling release kinetics, with $M_{t}$ the amount of drug released in time interval t, and $M_{\infty }$ the one released in infinite time interval, that will correspond, in fact, to the drug initially loaded into the polymer matrix.

The fact that, using proper approximations corresponding to the experimental conditions, the theoretical model can be reduced to a well-known equation describing the release kinetics, validates the theoretical model and confirms its hypothesis on the system evolution at microscopic level.

Through comparison, the Higuchi constant can be written:

(29) $$k_{H}\;=\;2\frac{\sigma^{1/2}}{d}$$

where σ can be assimilated to the diffusion coefficient σ ≡ D, and d is the thickness of the hydrogel film. Through this relation, the Higuchi constant $k_{H}$ is correlated with system characteristics, specific for each polymer matrix, eliminating thus its empirical character.

## 4.4. Results and Discussion on the Theoretical Model

As previously mentioned, one of the theoretical modeling aims was, besides obtaining information regarding the evolution of the system at a microscopic level, to determine system-specific characteristic quantities such as the diffusion coefficient, Higuchi constant (as measure of release rate), and fractal dimension (as measure of system complexity), and how these are related to each other.

Thus, the Higuchi constant was determined by fitting the experimental data, at small time scale, i.e., the burst release, with Equation (28) with correlation factors between 0.86 and 0.9 (Table 4). Further, the diffusion coefficients were calculated for all samples, taking into consideration Equation (29) and the samples’ thickness (Table 4).

**Figure 8:**
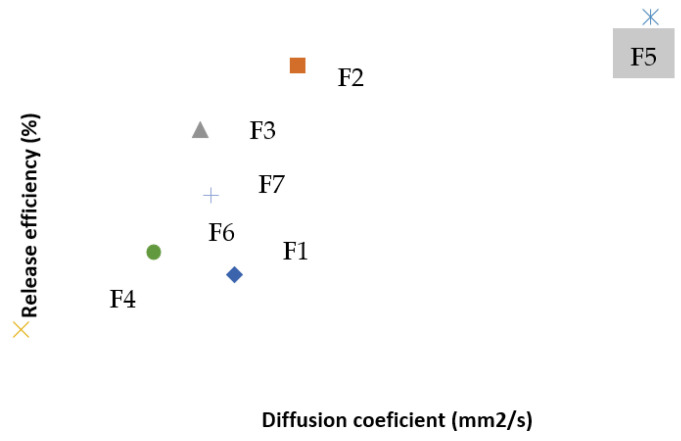
The correlation between the release efficiency and the diffusion coefficient.

A comparative analysis of these values, correlated with the experimental results from Section 2, shows that just the Higuchi constant values, alone, do not reflect the time evolution of swelling degree and release efficiency, related to the fact that sample F5 has the highest capacity of encapsulating and releasing, and, therefore, should have the higher Higuchi constant. Instead, the diffusion coefficients reflect, for most of the samples, their time evolution in terms of release efficiency (Figure 8), thus validating Equation (28) as a particular case of the proposed theoretical model.

**Figure 9:**
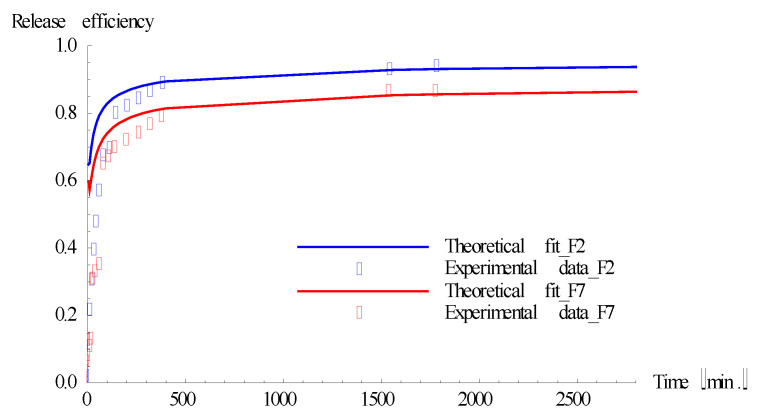
Theoretical and experimental release kinetics at long time scales.

For longer time scales, up to 2800 min, the release kinetics were analyzed through Equation (27), using the values of films’ thickness and diffusion coefficients from Table 4. It was found that the experimental values and theoretical ones are very well correlated for n = 2, suggesting that, at long time scales, such a solution becomes much more comprehensive than (28), taking into consideration the complexity of the processes behind the release dynamics. In Figure 9, representative experimental and theoretical release kinetics are represented.

We can conclude that the proposed theoretical model can characterize, through Equation (27), system evolution at different time scales of experimental observation, considering that longer time scales imply a higher complexity of the system, reflected in higher values for n.

## 5. Conclusions

In this study, a new type of hydrogel film based on kappa carrageenan with epichlorohydrin as a crosslinker agent was prepared and evaluated in order to determine its swelling and loading/release drug capacity. All release profiles demonstrated that the obtained hydrogel films can be promising and attractive materials for controlled drug delivery systems in a variety of applications.

The proposed theoretical model, based on the system evolution at microscopic level, allowed us to have a look” at what happens inside the drug delivery systems. Thus, it was observed that the complex system dynamics evolve from a normal period doubling state towards damped oscillating via strong modulated dynamics. The release kinetics are, as a matter of fact, the result of a constant overlapping of non-manifest and manifest release patterns. The non-manifest release pattern occurs at non-differentiable, i.e., microscopic scale, in the system background, and is hidden to the measurable values, but, on average, its cumulative effects impact the system evolution. The observable, measurable part of the drug release process, i.e., the amount of released drug, is, in fact, the result of the manifest release patterns. These patterns were manifested, at the macroscopic scale, through Fickian and non-Fickian diffusion processes. Moreover, the proposed theoretical model can characterize system evolution at different time scales of experimental observation, thus providing a comprehensive look to the entire release process.

## Figures and Tables

**Table 1:** Initial parameters for the synthesis of the films’ preparation and yields.

Sample Code	Concentration (%)	Molar Raport	βCD (g)	κCG (g)	NaOH (mL)	ECH (mL)	Yields (%)
F1	10	1:1	0.5	0.5	10	1.5	86.1
F2	15	1:2	0.5	1	10	1.5	68.1
F3	15	2:1	1	0.5	10	1.5	77.9
F4	6.6	1:1	0.5	0.5	15	1.5	25.9
F5	5	1:1	0.5	0.5	20	1.5	25.2
F6	10	1:1	0.5	0.5	10	0.75	71.0
F7	10	1:1	0.5	0.5	10	3	38.6

**Table 2:** Swelling degree—maximum values.

	F1	F2	F3	F4	F5	F6	F7
Swelling degree (%)	238	418	409	442	957	327	292

**Table 3:** Loaded and released amount of MT for the analyzed samples.

	F1	F2	F3	F4	F5	F6	F7
Loaded MT (mg)	12.6	16.2	13.3	16.1	17.9	16.2	10.5
Loaded Efficiency (%)	57.87	46.02	55.76	46.32	40.23	46.08	65.15
Released MT (mg/mL)	10.24	15.41	12.05	12.44	17.65	13.35	9.03
Released Efficiency MT (%)	80.99	95.13	90.80	77.25	98.42	82.53	86.39

**Table 4:** The Higuchi constant, the diffusion coefficients, and the fractal dimensions of all samples.

	$k_{H}\left(\min.^{-1/2}\right)$	d(mm)	$D\left(\frac{{\mathrm{mm}}^{2}}{s}\right)$
BCD-CG F1	4.13 × 10^−3^	7.55	4.051 × 10^−6^
BCD-CG F2	6.23 × 10^−3^	5.67	5.199 × 10^−6^
BCD-CG F3	5.48 × 10^−3^	5.22	3.421 × 10^−6^
BCD-CG F4	3.18 × 10^−3^	2.07	0.181 × 10^−6^
BCD-CG F5	5.77 × 10^−3^	9.14	11.58 × 10^−6^
BCD-CG F6	5.07 × 10^−3^	4.91	2.582 × 10^−6^
BCD-CG F7	5.44 × 10^−3^	5.42	3.622 × 10^−6^

## Data Availability

Not applicable.
